# Primary tracheal carcinoid tumor misdiagnosed as asthma: a rare case report

**DOI:** 10.1097/MS9.0000000000000388

**Published:** 2023-03-27

**Authors:** Oadi N. Shrateh, Afnan W.M. Jobran, Saja Jaber, Ahmad Kahla, Bashar S. Shamieh, Izzeddin A. Bakri, Yousef Abu Asbeh

**Affiliations:** a School of Medicine, Al-Quds University, Abu-Dis; Departments of b General Surgery; c Radiology; d Thoracic Surgery, Saint Joseph Hospital; e Faculty of Medicine, Al-Quds University; f Departement of Histopathology, Makassed Islamic Charitable Hospital, Jerusalem; g Department of Thoracic Surgery, Al-Ahli Hospital, Hebron

**Keywords:** tracheal, carcinoid, tumor, asthma, case report

## Abstract

About 2% of all lung malignancies are pulmonary carcinoid tumors, a family of neuroendocrine tumors. Rarely does a typical tracheal carcinoid of the trachea manifest as an endoluminal polypoidal tumor.

**Case presentation:**

The author describe a 61-year-old nonsmoker who complained of growing nonexertional shortness of breath 5 years ago. She also had a wheezy chest and a dry cough. The results of the chest radiography and electrocardiogram revealed no noteworthy abnormalities. The results of the pulmonary function test supported the diagnosis of bronchial asthma. A patient’s treatment has not advanced. After performing a bronchoscopy, a biopsy was taken and sent for pathological analysis. The endobronchial lining was found to have a subepithelial tumor infiltrate made up of nests of homogeneous bland cells with central nuclei and mild granular cytoplasm, according to histopathologic analysis. Considering all of these findings, the patient was diagnosed with a primary tracheal carcinoid tumor, which was misdiagnosed and treated as bronchial asthma.

**Discussion and conclusion:**

People with stridor or trepopnea symptoms should undergo a computed tomography scan since central airway tumors can mimic the symptoms of bronchial asthma while a chest radiograph may be normal. Tracheal carcinoid that has not progressed to the mediastinum can be successfully removed with flexible bronchoscopy and electrocautery, but the excision site needs to be continuously watched for recurrence.

## Introduction

Highlights

- The respiratory system’s endobronchial benign tumors are uncommon.
- Primary tracheal tumors are rare neoplasms that are difficult to detect on chest radiographs and have few symptoms, making them easy to miss.
- Since central airway tumors can mimic bronchial asthma symptoms and a chest radiography may be normal, individuals who exhibit stridor or trepopnea symptoms should have a further investigation, including a computed tomography scan and bronchoscopy.

The respiratory system’s endobronchial benign tumors are uncommon^1^,^2^. Carcinoids are among the most frequent benign endobronchial tumors; however, primary typical tracheal carcinoids are rare^1^,^2^. As a result of the sluggish rate of progression, bronchial asthma is sometimes misdiagnosed as having the same symptoms^3^. Not all of the patients may have hemoptysis. Trepopnea, which occurs when a patient has breathing problems in just one lateral decubitus posture, is an underdiagnosed type of dyspnea^3^. Primary tracheal tumors are uncommon neoplasms that are difficult to detect on chest radiographs and have few symptoms, making them easy to miss^3^. This case report has been reported in line with the SCARE Criteria^4^.

## Case presentation

Our patient is a 61-year-old nonsmoker female who was referred to us by a neighboring hospital. According to the patient’s medical records, she was admitted to the peripheral hospital 5 years ago for a complaint of progressive nonexertional shortness of breath associated with a wheezy chest and a dry cough. Electrocardiography and a chest radiography revealed unremarkable pathologies. The findings of the pulmonary function test were consistent with bronchial asthma (reduced forced expiratory volume in 1 s/forced vital capacity ratio <70% and significant bronchospasm reversibility was demonstrated by an increase in forced expiratory volume in 1 s of 12% and 200 ml postshort-acting bronchodilator administration). As a result, the patient was prescribed asthma medications and sent home.

After 1 year, the patient was referred to our hospital due to the worsening of the same symptoms without any significant improvement in her clinical condition, despite her bronchial asthma treatment regimen. The patient noticed a loss of weight of ~3 kg over the past 2 months, which was not associated with a decreased appetite. She denied any chest pain or discomfort, fever, chills, hemoptysis, difficulty swallowing, or regurgitation of food. Past medical history is unremarkable except for controlled mild hypertension. The patient reported no personal and/or family history of cancer; any acute, repeat, or discontinued medications; any allergies; any chronic or autoinflammatory diseases; any genetic or psychosocial issues; and a free past surgical history.

**Figure 1:**
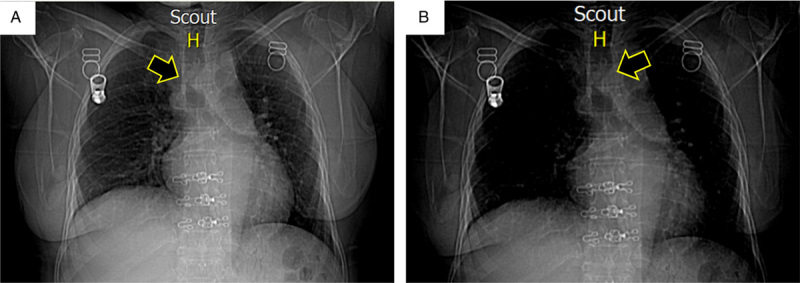
(A and B) A chest radiograph showing tracheal narrowing 2–3 cm above the carina (arrows).

**Figure 2:**
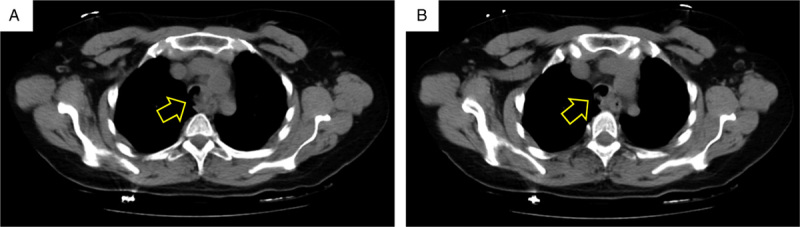
(A and B) A computed tomography scan of the chest showing endotracheal mass lesion narrowing the airway lumen (arrows).

**Figure 3:**
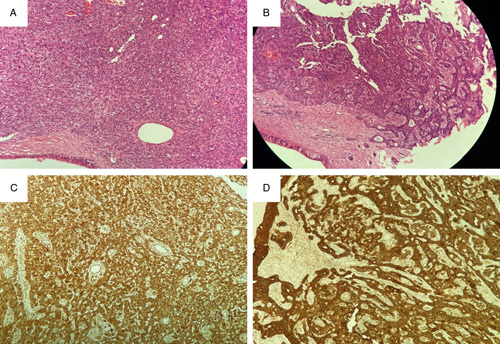
(A and B) A hematoxylin and eosin, ×10. (C) A positive NSE immunostain. (D) A positive pan-CK immunostain. NSE, neuron-specific enolase.

Upon admission, physical assessment revealed a mildly tachypneic patient with diffuse bilateral wheezing and expiratory stridor. Cardiac and abdominal examinations were noncontributory. Vital signs, electrocardiography, and laboratory evaluation, including a complete blood count and arterial blood gases, were normal. An radiography of the chest revealed a narrowing of the trachea about 2–3 cm above the carina (Fig. 1). A computed tomography (CT) scan of the chest showed a mass lesion in the lumen of the distal trachea (Fig. 2). A bronchoscopy was done, and a biopsy was obtained and sent for pathologic evaluation. Histopathologic examination revealed endobronchial lining with subepithelial tumor infiltrate composed of nests of uniform bland cells with central nuclei and moderate granular cytoplasm (Fig. 3A), cords and trabeculae of uniform bland cells with central nuclei and moderate granular cytoplasm (Fig. 3B), and positivity for neuron-specific enolase and pan-CK immunostains (Fig. 3C, D). A positron emission tomography (PET) scan was done and showed no evidence of a metabolically active malignant lesion elsewhere. Considering all of these findings, the patient was diagnosed with a primary tracheal carcinoid tumor, which was misdiagnosed and treated as bronchial asthma. Consequently, she underwent resection of the tracheal tumor with anastomosis. The procedure was performed by a consultant in cardiothoracic surgery at a private hospital. The patient then received 30 cycles of radiotherapy, with complete recovery of her symptoms. The patient was followed for 3 years, with CT scans and bronchoscopies every 6 months, and all tests revealed no tumor recurrence at the site of resection and a patent airway with normal mucosa. There were no readmissions to the hospital due to any respiratory or other complaints, and no other adverse events occurred.

## Discussion

Kulchitsky cells that are dispersed throughout the bronchopulmonary mucosa give birth to carcinoids^1^. The prevalence of these tumors, which make up around 2% of all bronchopulmonary cancers, is low^5^,^6^. Most bronchopulmonary carcinoids cells (75–90%) are restricted to the central airways, while the peripheral airways account for a smaller part (10–25%)^5^,^6^. Small cell carcinomas, large cell neuroendocrine carcinomas, and carcinoid tumors (typical and atypical) are the three primary subtypes of neuroendocrine tumors of the lung, respectively, according to WHO 2004^7^. Based on their appearance, mitotic rate, Ki-67 index, and presence of necrosis, these are further divided into three categories: well differentiated (low grade) typical, moderately differentiated (intermediate grade) atypical, and poorly differentiated (high grade) large cell neuroendocrine carcinomas and small cell carcinomas^7^.

Ninety percent of carcinoid lung neoplasms are typical carcinoid tumors, which primarily affect people under 40. Metastases to lymph nodes (5–15%) and distant locations (3%) are infrequent at presentation in patients with typical carcinoid tumors. Atypical carcinoids are less common than lung tumors (0.1–0.2%), but at the time of presentation, they frequently have distant (20%) or lymph node (40–50%) metastases^8^. According to Bagheri *et al*.’s^9^ findings, tracheal involvement was only detected in 5% of cases, while the left main bronchus was the most often affected region. As in our instance, tracheal carcinoids typically develop from the posterior noncartilaginous fibrous membrane in the distal portion of the trachea. While hemoptysis, wheezing, and dyspnea are the typical symptoms, our patient also displayed trepopnea, a rare symptom that has never been documented in the literature before. There is still a delay in the identification of primary tracheal tumors due to the trachea being a blind spot on the chest radiograph and the symptoms of wheeze and stridor being mistaken for asthma. While the ^18^F-FDG PET/CT scan has low sensitivity and specificity in separating the pulmonary carcinoids from other tumors, the ^68^Ga-DOTATOC PET/CT is an effective imaging investigation for the evaluation of pulmonary carcinoids with a sensitivity of 96% and 100% specificity^10^.

When circumscribed, carcinoid tumors are generally managed surgically. For normal and atypical carcinoid tumors, respectively, the 5-year survival rate following surgical excision is 97 and 78%^2^. The primary prognostic variables for these patients were their histology and lymph node involvement^2^. However, because metastatic tumors are typically resistant to chemotherapy or radiotherapy, there is not much to give patients with this condition.

Endobronchial tumor removal with bronchoscopy is a practical and effective method^1^,^6^,^11^. Endobronchial tumors that take up more than 50% of the major airways’ lumen and are linked to hemoptysis and dyspnea, lesions that inhibit mucociliary clearance and result in recurrent pneumonitis, or intractable coughs are all indications for endobronchial ablative therapy. Extrinsic airway compression is the only absolute contraindication to endobronchial ablative therapy. Endobronchial ablation may be performed with a flexible or rigid bronchoscope. Rigid bronchoscopy is the method of choice for obstruction removal in patients with severe airway obstruction, particularly in those who are experiencing respiratory failure. Ventilation can continue while concurrent airway procedures are being performed because of the ventilating rigid bronchoscope’s wide lumen. Endobronchial ablative techniques, such as laser, electrocautery, cryotherapy, and others^5^,^11^,^12^, can be used to remove endobronchial growths. These techniques include coring out the tumor using the rigid bronchoscope’s beveled tip.

## Conclusion

Since central airway tumors can mimic bronchial asthma symptoms and a chest radiography may be normal, individuals who exhibit stridor or trepopnea symptoms should have a CT scan performed. Flexible bronchoscopy and electrocautery can be used to successfully remove tracheal carcinoid that has not spread to the mediastinum, but the excision site needs to be closely monitored for recurrence.

## Ethical approval

Our institution has exempted this study from ethical review.

## Consent

A written informed consent for the data and picture was taken from the patient and the family and available upon request from the Editor-in-Chief.

## Sources of funding

This research did not receive any specific grant from funding agencies in the public, commercial, or not-for-profit sectors.

## Author contribution

O.N.S., A.W.M.J., S.J., and A.K.: writing the manuscript.

B.S.S. and I.A.B.: imaging description. O.N.S. and Y.A.A.: reviewing and editing the manuscript.

## Conflicts of interest disclosure

The authors declare that they have no known competing financial interests or personal relationships that could have appeared to influence the work reported in this paper.

## Research registration unique identifying number (UIN)

None.

## Guarantor

Oadi N. Shrateh.

## Provenance and peer review

Not commissioned, externally peer-reviewed.

## Authorship

All authors attest that they meet the current ICMJE criteria for authorship.

## References

[1] Gould VE Linnoila RI Memoli VA . Neuroendocrine components of the bronchopulmonary tract: hyperplasias, dysplasias, and neoplasms. Lab Investig 1983;49:519–537. 6138458

[2] García-Yuste M Matilla JM Cueto A . Typical and atypical carcinoid tumours: analysis of the experience of the Spanish Multi-Centric Study of Neuroendocrine Tumours of the Lung. Eur J Cardiothorac Surg 2007;31:192–197. 17196822 10.1016/j.ejcts.2006.11.031

[3] Asare AT Mensah F Acheampong S . Effects of gamma irradiation on agromorphological characteristics of okra (*Abelmoschus esculentus* L. Moench.). Adv Agric 2017;2017:7.

[4] Agha RA Franchi T Sohrabi C . The SCARE 2020 guideline: updating consensus surgical CAse REport (SCARE) guidelines. Int J Surg 2020;84:226–230. 33181358 10.1016/j.ijsu.2020.10.034

[5] Davini F Gonfiotti A Comin C . Typical and atypical carcinoid tumours: 20-year experience with 89 patients. J Cardiovasc Surg 2009;50:807. 19935614

[6] Rekhtman N . Neuroendocrine tumors of the lung: an update. Arch Pathol Lab Med 2010;134:1628–1638. 21043816 10.5858/2009-0583-RAR.1

[7] Travis WD . Pathology & genetics tumours of the lung, pleura, thymus and heart. World Health Organization Classific Tumours 2004;10:179–84.

[8] Gustafsson BI Kidd M Chan A . Bronchopulmonary neuroendocrine tumors. Cancer 2008;113:5–21. 18473355 10.1002/cncr.23542

[9] Bagheri R Mashhadi MT Haghi SZ . Tracheobronchopulmonary carcinoid tumors: analysis of 40 patients. Ann Thorac Cardiovasc Surg 2011;17:7–12. 21587121 10.5761/atcs.oa.08.01309

[10] Venkitaraman B Karunanithi S Kumar A . Role of 68Ga-DOTATOC PET/CT in initial evaluation of patients with suspected bronchopulmonary carcinoid. Eur J Nucl Med Mol Imaging 2014;41:856–864. 24435773 10.1007/s00259-013-2659-5

[11] Madan K Agarwal R Bal A . Bronchoscopic management of a rare benign endobronchial tumor. Rev Port Pneumol 2012;18:251–254. 22463875 10.1016/j.rppneu.2012.02.003

[12] Madan K Agarwal R Aggarwal AN . Therapeutic rigid bronchoscopy at a tertiary care center in North India: initial experience and systematic review of Indian literature. Lung India 2014;31:9. 24669075 10.4103/0970-2113.125887 PMC3960825

